# Correspondence

**Published:** 1869-06

**Authors:** 


### CORRESPONDENCE.

### ARTICLE VI.

*Baltimore, May 6th*, 1869.

Mr. Editor :—

At the last meeting of the Baltimore Pathological Society, Dr. H. R. Noel exhibited to the Society a human monstrosity. The specimen was that of a double headed mulatto child, having four arms, one body and one pair of lower limbs. The heads were well formed, and the necks and shoulders were normal, but the bodies coalesced immediately below shoulders and clavicles, the two sterni running into one, and the body down to the feet being single and of the natural size.

This rare specimen was from Mathews Co., Va., and a third child born at the same time was alive and doing well as were also both father and mother. Dr. Fitzhugh of Mathews Co. owns the specimen, and wishing to dispose of it, sent it to Baltimore and placed it under the care of Mr. Chas. Snead, of the firm of Thomas Norris & Son, opposite the Maltby House upon Pratt St.

Through the extreme kindness of Dr. W. E. Norris and the courtesy of Mr. Chas. Snead, Dr. Noel was made aware of the existence of this specimen and enabled to exhibit it before the Society. Mr. Snead thinks that it was born alive and cried some hours before it died.

